# What should we do about monkeypox?

**DOI:** 10.1002/ame2.12275

**Published:** 2022-09-23

**Authors:** Si Chen, Xue Li, Liying Zhang, Linzhu Ren

**Affiliations:** ^1^ College of Animal Sciences, Key Lab for Zoonoses Research, Ministry of Education Jilin University Changchun China

## Abstract

Monkeypox virus can infect several animals, including squirrels, Gambian poached rats, dormice, prairie dogs, monkeys, humans, etc. As reported, about 52 015 laboratory‐confirmed cases, including 18 deaths, have been reported to WHO from 102 member states across all 6 WHO regions from 1 Jan 2022 to 2 Sep 2022. WHO defined the disease as a Public Health Emergency of International Concern (PHEIC) on 21 July 2022. These data showed that monkeypox is novel global threat.

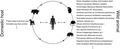

Over the past 3 years, the COVID‐19 pandemic has posed a significant global challenge to the public health system. However, other diseases, such as hepatitis of unknown origin in children and monkeypox, have been recently reported in European, African, and American countries. These data showed that monkeypox and other emerging or re‐emerging viruses are novel global threats.

Monkeypox is caused by infection with the monkeypox virus (MPXV), a linear, enveloped, double‐stranded DNA (dsDNA) virus belonging to the *Orthopoxvirus* genus in the subfamily *Chordopoxvirinae* of the family *Poxviridae*. There are 12 species in the *Orthopoxvirus* genus: Abatino macacapox virus, Akhmeta virus, camelpox virus, cowpox virus, ectromelia virus, monkeypox virus, raccoonpox virus, skunkpox virus, taterapox virus, vaccinia virus, variola virus, and volepox virus. Monkeypox is a zoonotic viral disease, which was first discovered in cynomolgus monkeys in 1958 and then reported in a human infection case in the Democratic Republic of the Congo (DRC) in 1970.^1^, ^2^ Since then, monkeypox has been endemic primarily in tropical rainforest areas of Central and West Africa and is currently widespread in Europe and North America.^2^, ^3^ Notably, from January 1, 2022, to July 23, 2022, more than 16 000 confirmed cases of monkeypox and five deaths in 75 non‐endemic countries and territories have been reported. In addition, these cases and their continuous transmission chains have been reported in countries with no direct or immediate epidemiological links with West or Central Africa. Thus, WHO defined the disease as a public health emergency of international concern (PHEIC) on July 23, 2022. As of September 14, 2022, about 59 147 laboratory‐confirmed cases, including 22 deaths, have been reported to WHO from 103 member states across all six WHO regions (https://worldhealthorg.shinyapps.io/mpx_global/). The number of weekly reported new cases globally increased by 3.2% in week 36 (September 05–September 11, *n* = 4863) compared to the previous week (*n* = 5026). Furthermore, WHO defines the global risk as moderate globally. However, WHO estimates the risk in the European region as high, and moderate in the African, Americas, Eastern Mediterranean, and Southeast Asia regions. The risk in the Western Pacific region is identified as low‐moderate (https://worldhealthorg.shinyapps.io/mpx_global/).

MPXV can infect several animals, including squirrels, Gambian poached rats, dormice, prairie dogs, monkeys, and humans.^2^, ^3^, ^4^, ^5^, ^6^ Human‐to‐human transmission through the air requires long‐term, face‐to‐face contact with the infected person. The virus can also be transmitted through direct contact with body fluids or lesions or indirect contact with diseased substances (e.g., clothes or bedding). Animal‐to‐human transmission can occur through bites or scratches, game processing, and direct or indirect contact with body fluids or lesions. The virus enters the human body through damaged skin, respiratory tract, or mucous membrane. Moreover, recent studies showed that the virus could be transmitted from humans to dogs.^7^ Therefore, because of its dissemination and harmfulness, it should be given equal importance to Avian influenza and COVID‐19.

The essential elements of the epidemic of infectious diseases include the pathogenic microorganisms (virus, bacterial, fungus, etc.), the route of transmission, and the susceptible people or animals. Recently, Bernstein et al. evaluated the cost and benefit of primary prevention of zoonotic infectious diseases.^8^ They found that primary pandemic prevention, including better monitoring of pathogen spillover, developing a global database of virus genomics and serology, better management of wildlife trade, and substantial reduction in deforestation, can save 19/20 of the cost of novel emerging viral zoonoses.^8^ The WHO defines five phases (levels) of infectious disease: pre‐emergence, emergence, localized transmission, epidemic, and pandemic. Bernstein et al. suggested adding a sixth phase, pathogen spillover, between the pre‐emergence and emergence phases.^8^ We totally agree. However, several other concerns or suggestions need to be considered.

First, for known pathogens, standard procedures of the five or six phases can be taken to control pathogens, cut off transmission routes, and protect susceptible people/animals. However, we cannot wait until the outbreak or severe infectious cases occur before taking appropriate measures. On the contrary, it is necessary to prevent and control the spread of novel pathogens or known pathogens causing severe diseases in advance according to a higher level of prevention and control strategies. For example, the prevention and control strategy adopted by WHO in COVID‐19 was inappropriate. At that time, human‐to‐human and cold‐chain transmission cases of SARS‐CoV‐2 had been reported in China,^5^, ^9^ but WHO still ignored these transmissions and failed to provide appropriate prevention and control strategies in time. The measures taken by WHO lagged the epidemic development speed, eventually leading to the epidemic's rapid spread without effective control. If we had taken higher‐level prevention and control measures for the COVID‐19 pandemic with reference to SARS‐CoV and MERS, it might have effectively stopped the spread of the pandemic. For these globally prevalent infectious diseases, the government's decision‐making, the masses' cooperation, theoretical research, and technical support are essential factors in overcoming and solving difficulties. Although blocking the spread of the virus will significantly affect the movement of people and flow of international trade, in the face of the concept of the same health, such losses and efforts are still worthwhile. Therefore, for the monkeypox epidemic that is evolving at present, there will be more confirmed cases in both endemic and non‐endemic countries, and it is necessary to take higher‐level measures for prevention and control of the disease because its current mortality rate is 1%–10%, or it will be higher with the development of the epidemic.

The second measure is to carry out widespread science propaganda to increase the public's awareness of diseases and their pathogens (including pathogens in the same genus) and improve their awareness of preventing infectious diseases. In the past 30 years, the monkeypox epidemic has spread only in some African countries. However, the recent sharp increase in monkeypox infection cases in non‐endemic countries indicates that the transmission ability of the virus in humans has improved. Monkeypox spreads through close contact, respiratory droplets, and sexual transmission. Although the smallpox vaccine shows high protective efficiency of about 85% against monkeypox, its protective effect will decrease with time. Notably, because smallpox was eradicated in 1980 and the smallpox vaccination program was terminated worldwide, most people have not been vaccinated with the smallpox vaccine. At the same time, the existing smallpox vaccines, modified vaccinia Ankara (Jynneos/Imamune/Imvanex, Bavarian Nordic, Hørsholm, Denmark) and ACAM2000 (Emergent BioSolutions, Gaithersburg, MD, USA), are limited, and it is impossible to complete vaccination in a short time. Meanwhile, the clinical symptoms of monkeypox are similar to those of chickenpox and measles, and monkeypox can also be coinfected with herpes simplex virus, HIV, syphilis, or other pathogens. In addition, it will take a lot of work and time to investigate animal sources and transmission routes. Therefore, it is necessary to let the public know about monkeypox and its infectivity and pathogenicity and master the methods to prevent the disease. Widespread science propaganda should be carried out in communities, medical stations, and other places where people live or gather, not just on the websites of WHO and the Centers for Disease Control and Prevention (CDC). In addition, people who contact animals should take necessary precautions, and the hunting and consumption of wild animals should be permanently banned.

The third measure is to minimize the spillover of monkeypox from animals, especially rodents and pets. Although monkeypox was first identified in Asian monkeys in Copenhagen in 1958, the MPXV has a broad spectrum of hosts. It is worth noting that except for rodents (e.g., squirrel, rats, and shrew species), monkeys, African hedgehogs, woodchucks, porcupines, giant anteaters, domestic pigs, and nonhuman primates (e.g., giant anteaters, orangutans, gorillas, chimpanzees, gibbons, and marmosets),^10^ the host spectrum or susceptible host of monkeypox is still unknown. As reported, the virus is transmitted from monkeys or rodents (squirrel, rat, and shrew species) to captive animals and pets (e.g., prairie dogs) and then to humans through close contact. In addition, recent evidence that MPXV could spread from human to human and animals (pet dogs) and cause disease suggests a need for animal safety and potential risk factors for animal transmission. In particular, whether companion animals, wild animals, city or rural stray animals may act as vectors of the virus needs to be clarified. Therefore, cutting off the transmission route and protecting susceptible people/animals are the most effective ways to control spillover. Meanwhile, vaccinating or feeding pets and wild animals with the poxvirus vaccine will also benefit disease control.

The fourth suggestion is that novel diagnostics, vaccines, and therapeutics are still immediately needed. Although several detection methods have been reported and validated,^11^, ^12^ Norz et al. found that the qPCR results from different specimen types in ambulatory and hospitalized patients infected with MPXV are various.^13^ The positive rate of qPCR in detecting lesion swabs caused by MPXV is reliable.^13^ In contrast, the false negative rates of qPCR in oropharyngeal swabs and blood samples are high,^13^ which may interfere with the diagnosis of the subclinical or asymptotic samples. Thus, a reliable method for MPXV diagnosis using multiple tissues or swabs is immediately needed. Furthermore, although the mutation rate of the virus is not as fast as that of the RNA virus (influenza virus, coronavirus, etc.), the current outbreak level and sequencing comparison results in Europe and America can still show that mutation is occurring or even worse. Therefore, it is also crucial to continuously monitor the mutation of the virus and develop fast and convenient diagnostic techniques that apply to the development of vaccines and drugs.

Moreover, although the smallpox vaccine can effectively inhibit monkeypox infection, the live vaccinia virus in the smallpox vaccine can cause rare but life‐threatening side effects, such as encephalitis and progressive vaccinia, especially in immunocompromised people. Therefore, it is important to evaluate the smallpox vaccine and *Orthopoxvirus* inhibitor in the nonhuman or animal model to explore their exact role in human MPXV infection. Besides, selecting suitable experimental animals is the key to determining the pathogenic mechanism of viruses and evaluating antivirals and vaccines. However, the reliability of replacing experimental animals must be re‐evaluated due to the shortage of experimental monkeys (e.g., Cynomolgus and Rhesus monkeys). Furthermore, human or animal health requires the cooperation and support of the whole society to create “One World, One Health.” Therefore, we should work together to control the endemic and build a community with a shared future for humankind.

## AUTHOR CONTRIBUTIONS

Conceptualization, Linzhu Ren; writing—original draft preparation, Si Chen and Linzhu Ren; writing—review, Linzhu Ren and Liying Zhang; revision, Si Chen and Xue Li; supervision, Linzhu Ren; and funding acquisition, Linzhu Ren. All authors have read and agreed to the published version of the manuscript.

## CONFLICT OF INTEREST

The authors declare that they have no competing interests. Linzhu Ren is an Editorial Board member of AMEM and a co‐author of this article. To minimize bias, he was excluded from all editorial decision‐making related to the acceptance of this article for publication.

## Data Availability

All data are available in the main text.
